# FoxM1 knockdown enhanced radiosensitivity of esophageal cancer by inducing apoptosis

**DOI:** 10.7150/jca.76671

**Published:** 2023-02-05

**Authors:** Qin Qin, Hui Chen, Huazhong Xu, Xiaowen Zhang, Junqiang Chen, Chi Zhang, Jia Liu, Liping Xu, Xinchen Sun

**Affiliations:** 1 Department of Radiation Oncology, the First Affiliated Hospital of Nanjing Medical University, Nanjing, China.; 2 Department of Neurosurgery, Sir Run Run Hospital, Nanjing Medical University, Nanjing, China.; 3 Department of Radiation Oncology, Fujian Cancer Hospital & Fujian Medical University Cancer Hospital, Fuzhou, China.

**Keywords:** FoxM1, esophageal cancer, radioresistance, apoptosis, IAPs

## Abstract

Radioresistance is a main reason for local recurrence of esophageal squamous cell carcinoma (ESCC). Forkhead box M1 (FoxM1) is implicated in cancer progression and chemoresistance. This study aims to determine the role of FoxM1 in ESCC radioresistance. We found that FoxM1 protein was upregulated in ESCC tissues compared with adjacent normal tissues. *In vitro* assays revealed that following irradiation, Eca-109, TE-13, and KYSE-150 cells had increased levels of FoxM1 protein. FoxM1 knockdown resulted in significantly reduced colony formation and increased cell apoptosis following irradiation. Moreover, FoxM1 knockdown induced ESCC cells to accumulate in the radiosensitive G2 /M phase and impeded the repair of radiation-induced DNA damage. Mechanistic studies indicated that radiosensitization of ESCC enhanced by FoxM1 knockdown was associated with increased BAX/BCL2 ratio as well as downregulated Survivin and XIAP, followed by the activation of both extrinsic and intrinsic apoptosis pathways. In xenograft mouse model, the combination of radiation and FoxM1-shRNA led to a synergistic anti-tumor effect. In conclusion, FoxM1 is a promising target to enhance radiosensitivity of ESCC.

## Introduction

In China and other Asian countries, esophageal squamous cell carcinoma (ESCC) is one of the most frequently diagnosed and lethal tumors of the gastrointestinal tract 1, 2. At the time of diagnosis, 60-70% patients with ESCC are surgically unresectable 3. Radiotherapy is one of the predominant treatment modalities for ESCC, especially locally advanced patients. Despite the advancement in radiotherapy, the mortality rate of ESCC is still high, and the long-term survival remains poor because of the resistance of ESCC cells to radiation 4-6. Therefore, the mechanisms that confer radiation resistance of ESCC cells must be elucidated.

Forkhead box M1 (FoxM1), a member of the forkhead superfamily of transcription factors, is normally expressed in embryonic tissues and proliferating adult tissues 7-9. FoxM1 plays a critical role in the regulation of a wide range of biological activities, including cell division, cell cycle progression, DNA damage repair, apoptosis, cell differentiation, and angiogenesis 8, 9. Abnormal expression of FoxM1 has been observed in malignant breast, liver, brain, prostate, lung, cervix, and pancreas cancer 10-14. Moreover, FoxM1 deregulation is involved in the chemoresistance of many cancer types, and the mechanism includes the activation of DNA damage repair pathway or oncogenic signaling mediated by MELK 15, 16. However, the role of FoxM1 in the radioresistance of ESCC remains unclear although recent evidence indicates the involvement of FoxM1 in the radioresistance of glioblastoma and lung cancer 12, 17. FoxM1 was reported to be overexpressed in ESCC tissue and correlated with poor prognosis of ESCC 18,19. Therefore, this study aimed to explore the role of FoxM1 in ESCC radioresistance and validate it as a radiosensitizing target in ESCC.

## Materials and Methods

### Tissue specimens

A total of 85 pairs of ESCC tissues and adjacent nontumorous tissues were obtained from 85 ESCC patients who had surgical resection of esophagus at the First Affiliated Hospital of Nanjing Medical University between 2012 and 2014. This study was approved by the Ethics Committee of the First Affiliated Hospital of Nanjing Medical University (2016-SRFA-074). All patients signed informed consents prior to their enrollment in this study.

### Immunohistochemical (IHC) analysis

Formalin-fixed and paraffin-embedded specimens were cut into 4 µm-thick sections for IHC. Then the tissue slides were de-paraffinized and rehydrated, followed by treatment with 3% hydrogen peroxide to block endogenous peroxidase activity. After incubation with 10% normal serum for 20 min, the slides were incubated with FoxM1 primary antibody (1:100; Santa Cruz Biotechnology, USA) overnight at 4°C. Subsequently, the slides were incubated for 30 min at 37°C with secondary antibody (1:5,000; Abcam, USA). Finally, the slides were stained with chromogen 3,3′-diaminobenzidine (DAB) and counter-stained with 0.1% hematoxylin.

According to the percentage of positive tumor cells, FoxM1 expression level was classified into five groups, 0, 0-5%; 1, 6%-25%; 2, 26%-50%; 3, 51%-75%; and 4, ≥76%. The intensity of immunostaining was scored as follows: 0, negative; 1, faint yellow; 2, pale brown; and 3, tan. Therefore, a weighted score was obtained by multiplying the percentage and intensity scores. Finally, FoxM1 staining was defined as low expression for the weighted score ≤6 points and high expression for the weighted score >6.

### Cell culture

ESCC cell lines Eca-109, TE-13, and KYSE-150 were cultured in RPM1-1640 medium (KeyGen Biotech, China) supplemented with 10% fetal bovine serum (FBS) (Gibco, USA) and 1% penicillin/streptomycin (Hyclone, USA) at 37°C in a humidified 5% carbon dioxide atmosphere.

### Gene silencing using RNAi

For transient gene silencing, cells were transfected with small interfering RNA (siRNA) targeting human *FoxM1* gene (GGCCCAACAAAUUCAUCCUTT) and control siRNA (UUCUCCGAACGUGUCACGUTT) synthesized by GenePharma (Shanghai, China) using Lipofectamine 2000 reagent (Invitrogen, USA).

FoxM1 short hairpin RNA (shRNA) (LV2-FoxM1-homo-586) and negative control lentiviral particles (LV2-NC) were purchased from GenePharma (Shanghai, China). A total of 4×10^5^ cells in exponential growth phase were plated into 6-well plates and cultured until 80% confluent. Then, the cells were transfected with 10 MOI of LV2-FoxM1-homo-586 or LV2-NC with 5 µg/ml polybrene. After 48 h, puromycin (10 µg/ml) was used to select the clones stably expressing shRNA. Western blot analysis was performed to determine the expression level of FoxM1 in selected clones.

### Colony formation assay

Exponentially growing cells were plated in six-well plates at a density of 200-5,000 cells/well. After culturing for 14 days, the cells were then fixed using methanol and stained with crystal violet. Clones composed of at least 50 cells were counted. The average data were fitted into the multitarget single-hit formula as follows: S=1-(1-e^-D/D0^)^N^. The survival enhancement ratio (SER) was calculated as follows: SER= Mean Inactivation Dose (control) / Mean Inactivation Dose (siRNA-treated).

### Western blot analysis

Western blot analysis was performed as previously described 20. Antibodies against FoxM1, survivin, XIAP, Cyclin B1, phospho-Cdc25c, Bax, Bcl-2, cleaved PARP, and cleaved caspase 3 were purchased from Cell Signaling Technology (Danvers, MA, USA), and antibodies against Ki67 and GAPDH were purchased from Santa Cruz Biotechnology (Dallas, TX, USA). The antibody against phospho-histone H2AX, Ser139 (γ-H2AX) was purchased from EMD Millipore (MA, USA).

### Apoptosis analysis

Apoptotic cells were detected by flow cytometry following staining with fluorescein-conjugated Annexin V-FITC/propidium iodide (PI) Kit (KeyGen Biotech, China) according to the manufacturer's instruction.

### Cell cycle analysis

Cells were fixed with ice-cold ethanol (70%) and incubated at -20°C overnight. After washing with PBS, the cells were resuspended in staining solution containing 20 µl of RNase A solution and 400 µl of PI staining solution (Vazyme biotech Co., Ltd.) and then subjected to a fluorescence-activated cell sorter (BD FACS Calibur) for cell cycle analysis.

### Immunofluorescence staining

Immunofluorescence staining of γ-H2AX foci was performed as previously described 20. Three random fields of each treatment group were captured under a confocal microscope (Leica). γ-H2AX positivity was defined as nuclei containing ≥10 immunoreactive foci.

### Xenograft tumor model

Sixteen male BALB/c nude mice (4-week-old, weight 18-20 g) were obtained from Nanjing Medical University Animal Center (Nanjing, China). Animal experiments were approved by Ethics Committee of Nanjing Medical University. The mice were randomly divided into 4 groups (n=4): 1) NC, infected with empty vector-encoded lentivirus; 2) FoxM1-shRNA; 3) NC+irradiation; and 4) FoxM1-shRNA+irradiation. Eca-109 cells (5×10^6^ cells in 0.1 ml of PBS) were injected subcutaneously into the right armpit of the mice. After 10 days, the transplanted tumors of mice were irradiated with a single dose of 8 Gy (4.48 Gy/min) using an RS-2000 biological irradiator. The diameter of the tumor and the body weight were measured every 2 days. Tumor volume was determined according to the following formula: (length[L] × width[W]^2^)/2. The mice were sacrificed on the 28th day, and the tumor weight was measured.

### TUNEL assay

Tumors were harvested and fixed in formalin. TUNEL assay was performed following the standard protocol as previously described 21.

### Statistical analysis

The association between expression of FoxM1 and histological type was evaluated by the χ2 test. Continuous data were expressed as the mean ± standard deviation from three independent experiments. Statistical significance was determined by Student's t-test. Statistical analysis was performed using SPSS version 17.0 software (SPSS, Inc., Chicago, IL, USA) or GraphPad Prism 6.0 software. Two-sided *p*-values < 0.05 were considered statistically significant.

## Results

### FoxM1 was overexpressed in ESCC tissue and upregulated in irradiated ESCC cells

**Figure 1:**
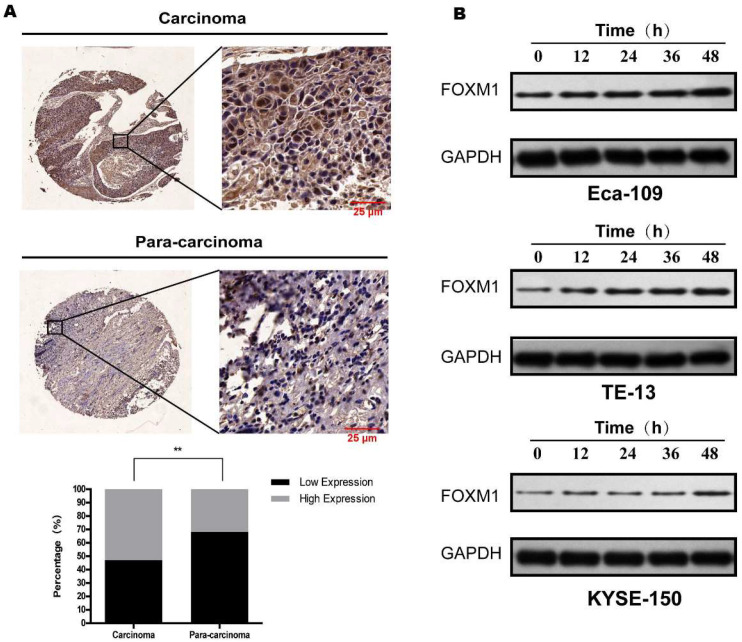
**FoxM1 is overexpressed in ESCC tissue and upregulated following irradiation** (A) IHC revealed remarkable stronger staining of FoxM1 in esophageal squamous cell carcinoma tissues compared with the para-carcinoma tissues (P<0.05). FoxM1 was mainly distributed in the cytoplasm of tumor cells. (B) Western blot analysis revealed that in all three ESCC cell lines, FoxM1 protein level was decreased over time.

Eighty-five pairs of ESCC cancerous and paracancerous tissue samples were evaluated for FoxM1 expression by IHC. As shown in Figure 1a, FoxM1 staining was located in the cytoplasm. FoxM1 expression in the cancerous tissue specimen (45/85, 52.9%) was significantly higher than in the paracancerous tissue (27/85, 31.8%; *p*=0.005) (Figure 1A).

Next, we detected FoxM1 levels in three ESCC cell lines after X-ray irradiation. As presented in Figure 1B, in all three cell lines, a single dose of irradiation increased the expression of FoxM1 after 12 h, and the elevated level was maintained at all time points, peaking at 48 h after irradiation. The radiation induced upregulation of FoxM1 in ESCC cells suggested that FoxM1 plays a critical role in radiation sensitivity of ESCC.

### FoxM1 knockdown sensitized ESCC cells to radiation

**Figure 2:**
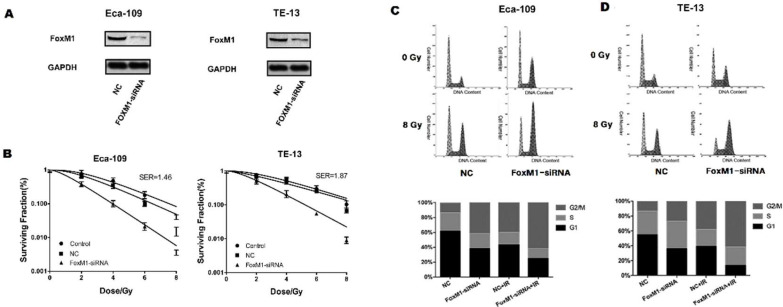
** FoxM1 knockdown sensitizes ESCC cells to radiation by inducing G2/M phase arrest.** (A) Western blot analysis demonstrated that FoxM1 protein was downregulated in both Eca-109 and TE-13 cells following siRNA transfection. (B) Cell survival curves showed that FoxM1-siRNA inhibited colony formation upon irradiation. The cells were transfected with saline, control-siRNA or FoxM1-siRNA in combination with ionizing radiation and harvested after 14 d culture. Each bar represented mean ± SE from 3 independent experiments. (C) FoxM1-siRNA transfection induced more Eca-109 cells arrested in G2/M phase following irradiation. (D) FoxM1-siRNA transfection induced more TE-13 cells arrested in G2/M phase following irradiation.

To assess the role of FoxM1 in the radiosensitivity of ESCC cell lines, we synthesized siRNA targeting FoxM1. In both Eca-109 and TE-13 cell lines, FoxM1-siRNA significantly reduced endogenous FoxM1 levels compared with control-siRNA (Figure 2A), resulting in decreased colony formation of ESCC cells after irradiation (Figure 2B). Significant differences in survival between FoxM1-siRNA and control-siRNA treated ESCC cells were observed at all four radiation doses. The sensitization enhancement ratios (SER) of Eca109 and TE-13 cells were 1.46 and 1.87, respectively. Table 1 summarized the radiobiological variables. The knockdown of FoxM1 radiosensitized ESCC cell lines, suggesting that FoxM1 may promote ESCC radioresistance.

### FoxM1 knockdown promoted ESCC cell accumulation at the G2/M phase

Cytometry analysis was conducted to evaluate the impact of FoxM1 knockdown on the ESCC cell cycle distribution before and after irradiation. The proportion of G2/M phase cells in the FoxM1-siRNA group was significantly higher than that in the control group, increasing from 12.9% to 41.3% in Eca-109 cells and from 13.9% to 27.1% in TE-13 cells. This effect remained evident after 8 Gy irradiation (Eca-109: 39.8% vs. 61.6%; TE-13: 38.1% vs. 61.6%) (Figure 2C, D). FoxM1 knockdown caused proliferating ESCC cells to accumulate in the radiosensitive G2 and M phases.

### FoxM1 knockdown impaired DNA damage repair of irradiated ESCC cells

**Figure 3:**
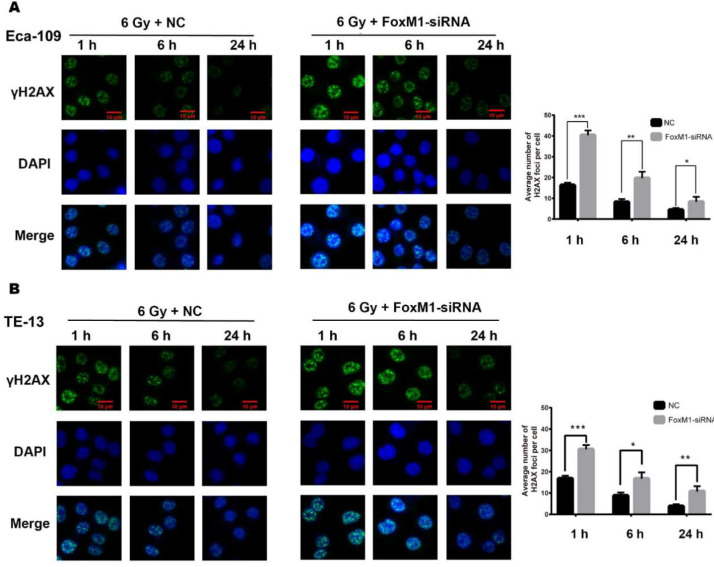
** FoxM1 knockdown inhibited DNA damage repair** (A) Immunfluorescence staining revealed significantly increased γ-H2AX foci in FoxM1 knockdown Eca-109 cells at all time points (1hr, 6hr, and 24hr) following 6Gy irradiation. (B) FoxM1-siRNA transfection also increased γ-H2AX foci in TE-13 cells. Bars represent average ± s.d. *p < 0.05, **p < 0.01 and ***p < 0.001.

FoxM1 is involved in the repair of DNA double strand break (DSB) after genotoxic drug treatment 22. Therefore, we evaluated the effect of FoxM1 knockdown on radiation-induced DNA damage response in Eca-109 and TE-13 cells. Indicative of DSB, γ-H2AX foci were detected at three time points (1, 6, and 24 h) after IR. As shown in Figure 3A, in the NC group of Eca-109, the number of γ-H2AX foci was significantly increased 1 h after irradiation (16.32±1.2). The DSBs were eventually repaired, and γ-H2AX signal decreased to the basal level at 24 h after IR (4.44±0.87). Compared with the NC group, a significant increase in γ-H2AX foci was observed in the FoxM1-siRNA group at all time points following IR (1 h, 40.32±2.3; 6 h, 19.65±3.1; 24 h, 8.33±2.4, *p*<0.05). Similar results were observed in TE-13 cells (Figure 3B). The strong γ-H2AX signal in ESCC cells transfected with FoxM1-siRNA indicated that FoxM1 knockdown inhibited the repair of radiation-induced DSBs.

### FoxM1 knockdown enhanced radiation-induced apoptosis of ESCC cells

**Figure 4:**
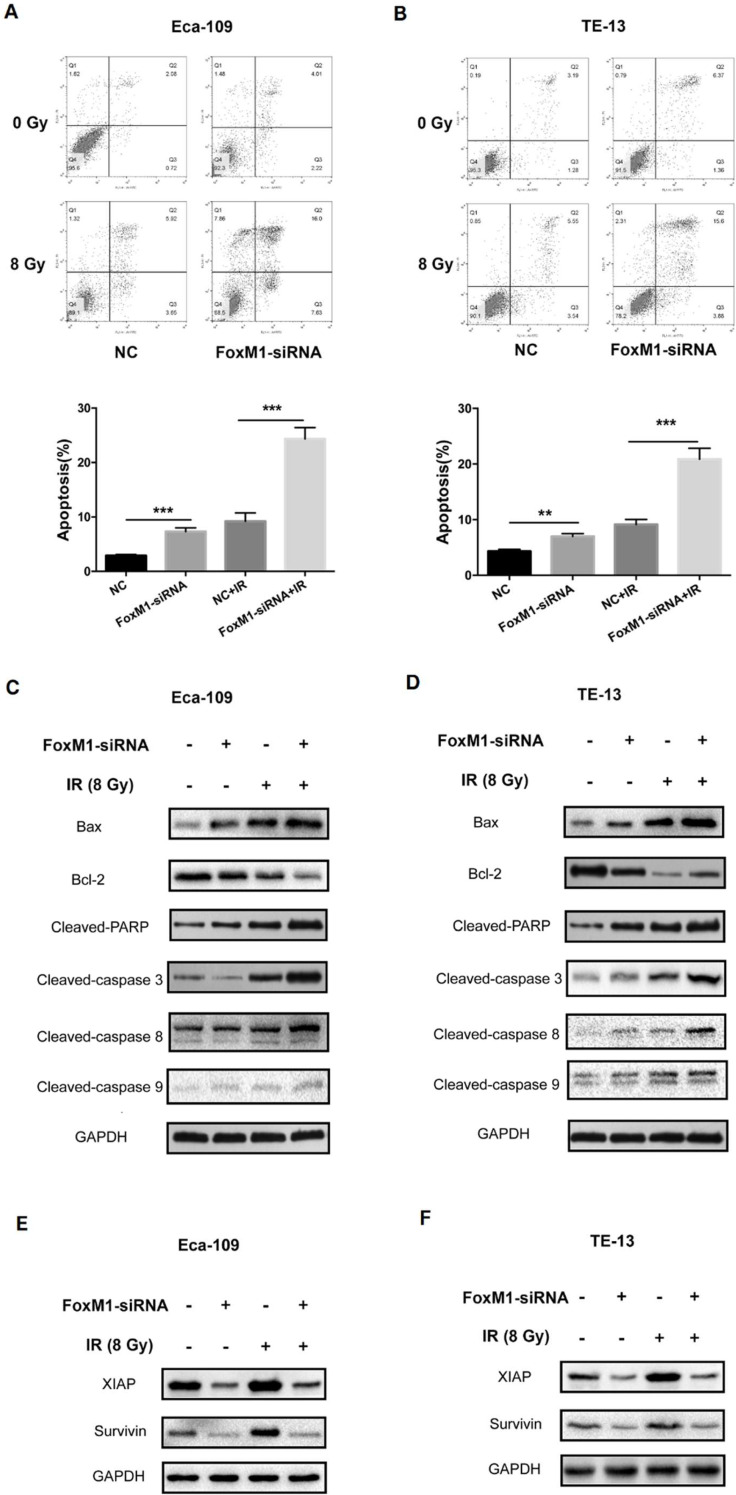
** FoxM1 knockdown enhances radiation induced apoptosis** (A, B) FoxM1-siRNA increased apoptosis ratio compared with control-siRNA and enhanced radiation induced cell apoptosis in both Eca-109 and TE-13 cells. (C, D) Western blot analysis of apoptosis proteins in Eca-109 and TE-13 cells. (E, F) Western blot analysis showed that knockdown of FoxM1 inhibited the expression of XIAP and Survivin proteins in Eca-109 and TE-13 cells. Bars represent average ± s.d. **p < 0.01 and ***p< 0.001. NC: Non-targeting control.

Flow cytometry was performed to quantify the percentage of apoptotic cells. As shown in Figure 4A, the apoptotic percentage of Eca-109 cell was 7.32±0.51% and 9.21±0.88% in the FoxM1-siRNA group and IR group, respectively. The combination treatment of FoxM1-siRNA and IR resulted in a significantly higher level of apoptosis (24.33±1.97%) in Eca-109 compared with either treatment alone (*p*<0.05). An increase in cell apoptosis was also observed in TE-13 cells after treatment with FoxM1-siRNA and IR (Figure 4B).

The alteration of BAX/BCL2 ratio and the activation of the caspase cascade are important for mitochondrial apoptosis pathway. As shown in Figure 4C,D, FoxM1 knockdown or IR alone led to an increase in the expression of pro-apoptotic BAX and a decline in the expression of anti-apoptotic BCL2 compared with the control. Notably, the combination of FoxM1 knockdown and IR resulted in a significant increase in BAX/BCL2 ratio. In both ESCC cell lines, FoxM1 knockdown in combination with IR led to stronger cleavage of caspase-3 and PARP compared with individual treatment. We detected the level of cleaved-caspase 8, a marker for the activation of the extrinsic apoptotic pathway, and cleaved-caspase 9, a marker for the intrinsic pathway. Treatment with either FoxM1-siRNA or IR resulted in slightly elevated level of caspase 8 or caspase 9 cleavages in Eca-109 and TE-13 cells. Combined treatment with FoxM1-siRNA and IR resulted in a higher level of cleaved-caspase 8 compared with either treatment alone (Figure 4C,D). FoxM1 depletion can enhance radiation-induced apoptosis by increasing the BAX/BCL2 ratio and inducing the activation of extrinsic apoptosis pathway.

Survivin and XIAP are two vital anti-apoptotic proteins. Nestal *et al.* confirmed the chemoresistance phenotype of FoxM1-overexpressing breast cancer cells associated with the upregulation of survivin and XIAP 23. We evaluated the effect of FoxM1 knockdown on survivin or XIAP expression in ESCC cells upon irradiation. As shown in Figure 4E,F, the expression levels of survivin and XIAP were elevated upon irradiation in the two ESCC cell lines. Survivin and XIAP levels were downregulated in FoxM1-inhibited ESCC cells. FoxM1 knockdown attenuated survivin and XIAP upregulation in response to irradiation. These data indicated that FoxM1 knockdown by siRNA might sensitize ESCC cells to radiation via the downregulation of survivin and XIAP.

### FoxM1 knockdown improved the efficacy of irradiation in ESCC xenograft tumor models

**Figure 5:**
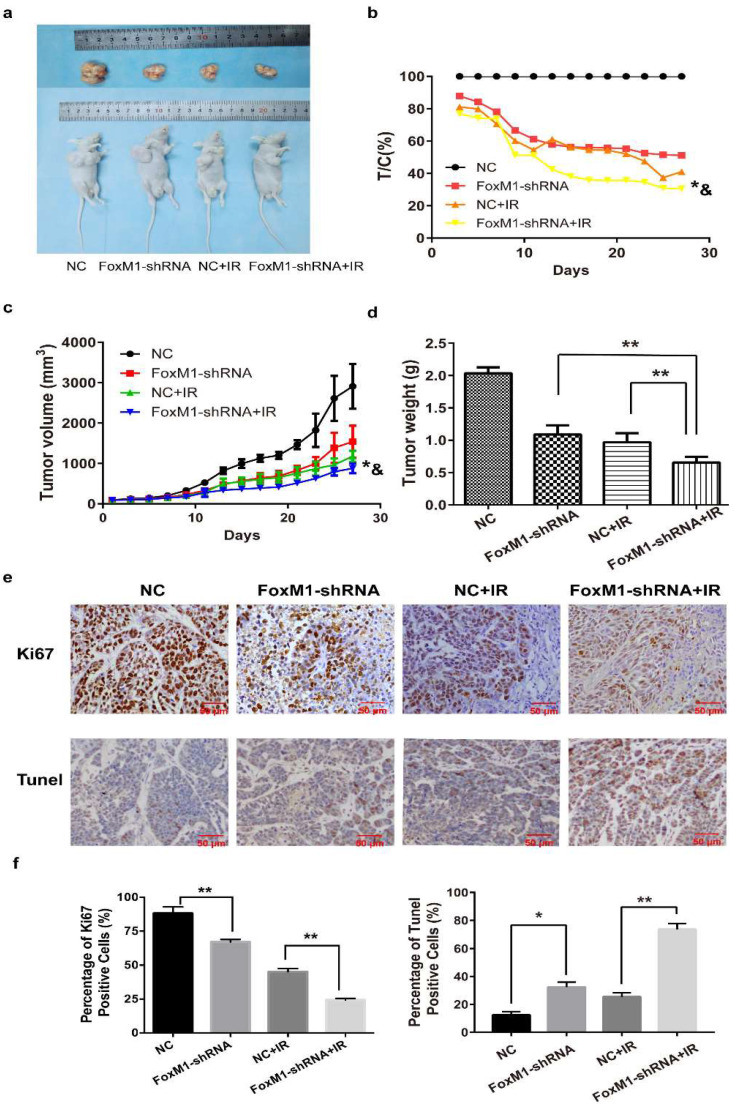
** FoxM1 knockdown enhances radiosensitivity of ESCC *in viv*o** (A) FoxM1 knockdown markedly inhibited growth of Eca-109 xenograft following irradiation in nude mice. (B) and (C) Tumor volume curves. * p=0.020 vs. FoxM1-shRNA; &* p*= 0.017 vs. NC+IR. (D) The combination treatment significantly inhibited the xenograft weight. **p < 0.01 (E) IHC analysis of Ki67 and TUNEL assay in Eca-109 tumor xenograft. (F) The histogram indicated the percentages of Ki67 positive cells and TUNEL positive cells in each treatment group. *p < 0.05; **p < 0.01.

Treatment with FoxM1-shRNA or IR alone moderately reduced tumor burden and weight in mice bearing Eca-109 subcutaneous tumors compared with the NC group (FoxM1-shRNA: T/C%= 51.1%; NC+IR: T/C%=41.1%) (Figure 5A,B). However, tumor growth inhibition was better when FoxM1-shRNA was combined with IR (FoxM1-shRNA+IR: T/C%=30.5%; vs. NC *p*=0.00038; vs. FoxM1-shRNA* p*=0.020; vs. NC+IR* p*= 0.017) (Figure 5C). Antitumor growth response, which was measured as change in mean tumor weight, indicated an additive effect of the combined FoxM1-shRNA and IR on tumor growth inhibition (vs. FoxM1-shRNA *p*= 0.002; vs. NC+IR* p*= 0.009) (Figure 5D). Compared with the NC group, no significant change was observed in the body weight of mice in all three treatment groups.

IHC analysis indicated that Ki-67 level was markedly lower in tumors treated with FoxM1-shRNA in combination with IR (percentage of Ki-67 positive cells: 24.38±1.12%) than in those treated with either FoxM1-shRNA (67.18±1.88%) or IR (45.18±2.32%) (Figure 5E,F). TUNEL assay revealed the significant increase in apoptotic cell percentage in tumor tissues from the combination treatment group (73.53±4.24%) compared with FoxM1-shRNA only (32.34±3.60%) or IR only group (25.37±2.94%) (Figure 5E,F).

## Discussion

The development of acquired radioresistance greatly limits the therapeutic benefit of patients with ESCC. FoxM1, which serves as a transcription factor for a wide range of genes, is associated with chemotherapy resistance, metastasis, and poor prognosis in a great number of malignancies. In esophageal cancer, elevated FoxM1 expression is correlated with the malignant progression of ESCC cells and poor outcome in patients who undergo curative resection 18,19. However, the involvement of FoxM1 in chemo- or radioresistance of ESCC is still poorly understood. It was reported that FoxM1 inhibition sensitized GBM cells to radiation 24. Given that irradiation induced the upregulation of FoxM1 in the surviving ESCC cells in a time-dependent manner, FoxM1 was postulated to be implicated in the radiation resistance of ESCC. Therefore, in the present study, we sought to determine the role of FoxM1 in the response of ESCC to radiation.

As anticipated, FoxM1 knockdown by RNA interference significantly suppressed colony formation of ESCC cell lines and impeded the growth of ESCC xenograft after irradiation, indicating the radiosensitizing effect of FoxM1 knockdown on ESCC both *in vitro* and* in vivo*.

FoxM1 plays an essential role in the regulation of G1-S and G2-M progression 9. A recent study showed that FoxM1 mediated ESCC cell proliferation by regulating CDC6 expression, which may promote G1-S phase transition of cell cycle 25. Pharmacological inhibition of FoxM1 induces G2/M arrest in papillary thyroid carcinoma cells and FoxM1-knockdown-induced cell cycle arrest in G2/M and subsequent apoptosis of GBM cells 24, 26. Consistently, this study showed that FoxM1 inhibition by siRNA led to the accumulation of ESCC cells at the G2/M phase, during which cancer cells are most sensitive to radiation. In concordance with diminished ESCC colony formation, FoxM1 knockdown combined with IR resulted in evident increase in the G2/M cell population. The involvement of FoxM1 in cell cycle regulation might contribute to ESCC radioresistance. FoxM1 also plays a role in many aspects of DNA damage response 27. Our study revealed that FoxM1 depletion significantly attenuated the DSB repair efficacy of irradiated ESCC cells as evidenced by the maintenance of γ-H2AX signal to more than 24 h post irradiation.

As a result of the increased cell proportion of G2/M phase and the attenuated capacity of DNA damage repair, treatment with FoxM1-siRNA enhanced the apoptosis of irradiated ESCC cells. Western blot analysis showed that FoxM1 knockdown or radiation alone caused the upregulation of Bax and the downregulation of Bcl-2 in ESCC cell lines. The combination of the two modalities demonstrated significant effects. Elevated BAX/BCL2 ratio triggers the loss of mitochondrial membrane potential followed by the activation of mitochondrial apoptosis pathway and caspase cascade 28. Our results showed that the combination of radiation with FoxM1-siRNA significantly increased caspase-3 and PARP cleavages. Moreover, both caspase-9 and caspase-8 were activated by the combination treatment, indicating that mitochondria-mediated apoptosis pathway and extrinsic apoptosis pathway participate in FoxM1 knockdown induced radiosensitivity of ESCC.

Inhibitor of apoptosis proteins (IAPs) inhibit caspase activity and protect cells from apoptosis 29,30. Our previous studies demonstrated that IAPs are involved in the ESCC apoptotic resistance to radiation. FoxM1 could target XIAP and survivin to promote cell growth and chemoresistance of breast cancer 23. The present study showed that the increased expression of XIAP and survivin in response to irradiation was significantly suppressed by FoxM1-siRNA, accompanied by attenuation of caspase activation. XIAP and survivin overexpression might contribute to FoxM1-mediated apoptotic resistance upon radiation in ESCC.

In corroboration with the *in vitro* results, the *in vivo* study of murine xenograft model revealed that FoxM1 suppression by shRNA significantly impeded tumor growth, which was further enhanced when combined with radiotherapy. The synergetic effect on ESCC growth inhibition is associated with the markedly decreased Ki-67 and increased TUNEL-positive cells, which suggested proliferation inhibition and apoptosis induction in ESCC cells.

In conclusion, both* in vitro* and* in vivo* findings suggested that FoxM1 knockdown sensitized ESCC cells to radiotherapy by impairing DNA damage response, arresting cell in G2/M phase, and enhancing cell apoptosis. Furthermore, XIAP and survivin downregulation as a result of FoxM1 knockdown partially explains the radiation resistance of FoxM1-proficient ESCC cells. To our knowledge, this is the first study to suggest that FoxM1 is a potential target to enhance radiosensitivity of ESCC.

## Figures and Tables

**Table 1:** Radiosensitizaion effects of FoxM1-siRNA on ESCC cell lines

	D_0_	Dq	SF2 †	SER ‡
**Eca-109**				
Control	2.20	2.50	0.80	1
Control-siRNA	2.05	1.84	0.69	1.07 1
FoxM1-siRNA	1.40	0.78	0.38	1.57 1.46
**TE-13**				
Control	3.01	2.53	0.81	1
Control-siRNA	3.37	1.39	0.70	0.89 1
FoxM1-siRNA	1.80	1.08	0.52	1.67 1.87

†SF2: Surviving fraction at 2 Gy; SER ‡: Sensitization enhancement ratio.
